# Development of a deep learning-based automated diagnostic system (DLADS) for classifying mammographic lesions — a first large-scale multi-institutional clinical trial in Japan

**DOI:** 10.1007/s12282-025-01741-3

**Published:** 2025-07-03

**Authors:** Takeshi Yamaguchi, Yoichi Koyama, Kenichi Inoue, Kanako Ban, Koichi Hirokaga, Yuka Kujiraoka, Yuko Okanami, Norimitsu Shinohara, Hiroko Tsunoda, Takayoshi Uematsu, Hirofumi Mukai

**Affiliations:** 1 Department of Medical Oncology, Toranomon Hospital, Tokyo, Japan; 2 Department of Breast Surgical Oncology, Tokyo Medical University Hospital, 6-7-1 Nishishinjuku, Shinjuku-Ku, Tokyo 160-0023 Japan; 3 Breast Cancer Center, Shonan Memorial Hospital, Kanagawa, Japan; 4 Yotsuya Medical Cube, Tokyo, Japan; 5 Department of Breast Surgery, Hyogo Cancer Center, Hyogo, Japan; 6 Department of Radiology, Tsukuba Memorial Hospital, Ibaraki, Japan; 7 Yuko Breast Clinic Meieki, Aichi, Japan; 8 Department of Radiological Technology, Faculty of Health Sciences, Gifu University of Medical Science, Gifu, Japan; 9 Department of Radiology, St. Luke’s International Hospital, Tokyo, Japan; 10 Department of Breast Imaging and Breast Interventional Radiology, Shizuoka Cancer Center Hospital, Shizuoka, Japan; 11 Division of Medical Oncology, National Cancer Center Hospital East, Chiba, Japan

**Keywords:** Artificial intelligence, Convolutional neural network, Breast cancer, Mammography, Computer-aided detection, Computer-aided diagnosis

## Abstract

**Background:**

Recently, western countries have built evidence on mammographic artificial Intelligence—computer-aided diagnosis (AI-CADx) systems; however, their effectiveness has not yet been sufficiently validated in Japanese women. In this study, we aimed to establish a Japanese mammographic AI-CADx system for the first time.

**Methods:**

We retrospectively collected screening or diagnostic mammograms from 63 institutions in Japan. We then randomly divided the images into training, validation, and test datasets in a balanced ratio of 8:1:1 on a case-level basis. The gold standard of annotation for the AI-CADx system is mammographic findings based on pathologic references. The AI-CADx system was developed using SE-ResNet modules and a sliding window algorithm. A cut-off concentration gradient of the heatmap image was set at 15%. The AI-CADx system was considered accurate if it detected the presence of a malignant lesion in a breast cancer mammogram. The primary endpoint of the AI-CADx system was defined as a sensitivity and specificity of over 80% for breast cancer diagnosis in the test dataset.

**Results:**

We collected 20,638 mammograms from 11,450 Japanese women with a median age of 55 years. The mammograms included 5019 breast cancer (24.3%), 5026 benign (24.4%), and 10,593 normal (51.3%) mammograms. In the test dataset of 2059 mammograms, the AI-CADx system achieved a sensitivity of 83.5% and a specificity of 84.7% for breast cancer diagnosis. The AUC in the test dataset was 0.841 (DeLong 95% CI; 0.822–0.859). The Accuracy was almost consistent independent of breast density, mammographic findings, type of cancer, and mammography vendors (AUC (range); 0.639–0.906).

**Conclusions:**

The developed Japanese mammographic AI-CADx system diagnosed breast cancer with a pre-specified sensitivity and specificity. We are planning a prospective study to validate the breast cancer diagnostic performance of Japanese physicians using this AI-CADx system as a second reader.

**Trial registration:**

UMIN, trial number UMIN000039009. Registered 26 December 2019, https://www.umin.ac.jp/ctr/

**Supplementary Information:**

The online version contains supplementary material available at 10.1007/s12282-025-01741-3.

## Introduction

To date, randomised controlled trials in Western countries have demonstrated a reduction in breast cancer-specific mortality due to screening mammography programs [1–3]. In Japan, screening mammography is recommended every two years for women aged 40 years and older [4]. Japanese screening mammograms are generally interpreted by two human readers (double reading), which may increase the cancer detection rate [5]. However, this system may require more human resources [5].

The breast cancer screening rate in Japan was 47.4% in 2022 (National Cancer Center, Cancer Statistics; https://ganjoho.jp/reg_stat/index.html), lower than over 70% in Western countries (OECD Health Statistics; https://www.oecd.org/en/data/datasets/oecd-health-statistics.html). Various efforts have been made to increase this rate in Japan; however, the increased number of women being screened and the use of double reading in screening mammography programs creates a high workload for readers. In addition, the diagnosis of mammograms is influenced by the experience of the readers involved in breast imaging [6, 7]. Therefore, alternative strategies are needed to reduce the burden, and implement screening mammography efficiently.

Mammographic computer-aided detection (CADe) systems have been developed to assist in the detection of breast cancer when reading mammograms. However, the clinical utility of traditional mammographic CADe systems, which are based on proprietary vendor algorithms, is not well-established. While some studies have reported improved reading performance [8–11], others have shown increased recall rates and unnecessary biopsies [12–14].

In contrast, the field of artificial intelligence (AI) is developing rapidly, and novel AI-CADe and AI-computer-aided diagnosis (CADx) systems have been developed using deep learning convolutional neural networks (CNN), some of which have already shown promising results [15–18]. With comparable capabilities to humans, AI-CADe and AI-CADx systems can help readers improve breast cancer diagnostic performance, and reduce workload for readers by pre-selecting suspicious lesions in mammography. However, these AI algorithms were mainly developed in Western countries, and their effectiveness has not yet been sufficiently validated in Japanese women. Since Asian women characteristically have higher dense breasts than women from other ethnic groups [19–21]. Furthermore, mammographic AI-CADe and AI-CADx systems are not widely used in daily clinical practice in Japan, indicating a stage of unmet medical need. Although the current clinical practice guidelines of the Japanese Breast Cancer Society suggest its usefulness, it is considered the future research question [4]. In this study, the AI-CADx system was developed using imaging data from Japanese women for the use in breast cancer screening in Japan.

## Materials and methods

This study was approved by the institutional review board of the National Cancer Center Hospital East (approval number: 2019–048), and the requirement for informed consent was waived according to Japanese clinical research guidelines and regulations.

### Mammograms

A total of 63 institutions, including hospitals and clinics in Japan participated in this study. Mammograms taken for breast cancer screening and diagnosis from January 2010 to August 2019 were collected retrospectively. All mammograms were either de-identified digital radiography mammograms or computed radiography mammograms. A mammogram included MLO (mediolateral oblique) and CC (craniocaudal) views. If not available, only one pair of MLO views was collected.

Mammograms were acquired using mammography equipment from several manufacturers. The most commonly used devices and manufacturers were Selenia-Dimensions (Hologic, USA) with 39.8%, AMULET-Innovality, AMULET-f (FUJIFILM, Japan) with 29.3%, MAMMOMAT-Inspiration, MAMMOMAT-Revelation (Siemens Healthineers, Germany) with 18.4%, and Senographe-CrystalNova, Senographe-Pristina, Senographe-Essential (GE HealthCare Technologies, USA) with 9.1%.

Mammograms were retrieved from the devices in uncompressed DICOM format and converted to PNG format for anonymization and data handling. The matrix size was consistent with the DICOM data retrieved from the device, and the grayscale was converted to 8 bits.

### Inclusion criteria

All mammograms were from Japanese women over 20 years of age, with no previous history of chemotherapy, endocrine therapy, or radiotherapy, and no history of surgery, including partial resection, breast reconstruction, incisional or vacuum-assisted biopsy, and mammoplasty. Eligible mammograms showed either only benign, only breast cancer lesions, or normal findings. Even if there are multiple lesions within the same mammogram, it is eligible if all of them can be detected and meet the following criteria;

-All breast cancer lesions were histologically confirmed. Atypical epithelial lesions, e.g., atypical ductal hyperplasia, and malignant mesenchymal tumors, e.g., malignant phyllodes tumor, were excluded.
-Lesions were classified as benign if one of the following criteria was met: (i) histologically proven as benign, (ii) no progression on separate mammograms performed at least 2 years apart, (iii) clear evidence of a simple cyst on mammography or other modalities.
-Normal breast mammograms meet one of the following criteria: (i) no lesions detected by mammography, ultrasound, and/or magnetic resonance imaging, (ii) no new appearance of lesions on separate mammograms taken at least 2 years apart.

### Exclusion criteria

The following cases were excluded from this study: (i) tomosynthesis, spot compression, synthetic 2-dimensional mammography, and poor quality images, (ii) images showing axillary lymph node metastases. (iii) cases of breast cancer or benign disease with no mammography findings when assessed by the investigating physicians. (iv) cases where breast cancer and benign lesions coexist in the same mammogram. (v) mammograms with quality degradation when converted to an irreversible image, i.e., JPEG format.

### Dataset

Based on the design of previous studies [22], we set the target number of mammograms to be collected at 20,000 mammograms. The expected number of breast cancer, benign, and normal mammograms was 5000, 5000, and 10,000, respectively.

Local investigators (a total of **72** readers ranked graded **A** (indicating breast cancer detection sensitivity and specificity greater than 90%), or equivalent to grade A, according to the Japan Central Organization on Quality Assurance of Breast Cancer Screening [https://www.qabcs.or.jp/]) manually marked all benign and breast cancer lesions based on pathology reports using Fiji (Image-J) software (https://fiji.sc/) (**Supplementary Fig. 1**). Benign cases without pathology reports were marked with reference to other modalities, e.g., ultrasound, and/or magnetic resonance imaging. The accuracy of the marking was finally verified by the authors (KI, KB, KH, YK, YO, NS, HT, TU).

After collecting eligible images, we randomly divided into a training, validation, and test datasets in a balanced ratio of 8:1:1 on a case-level basis. No adjustment was made for this randomization. The training and validation datasets were used to build and refine the AI-CADx system. The test dataset was used to evaluate the final AI-CADx system.

### Construction of an AI algorithm

**Fig. 1:**
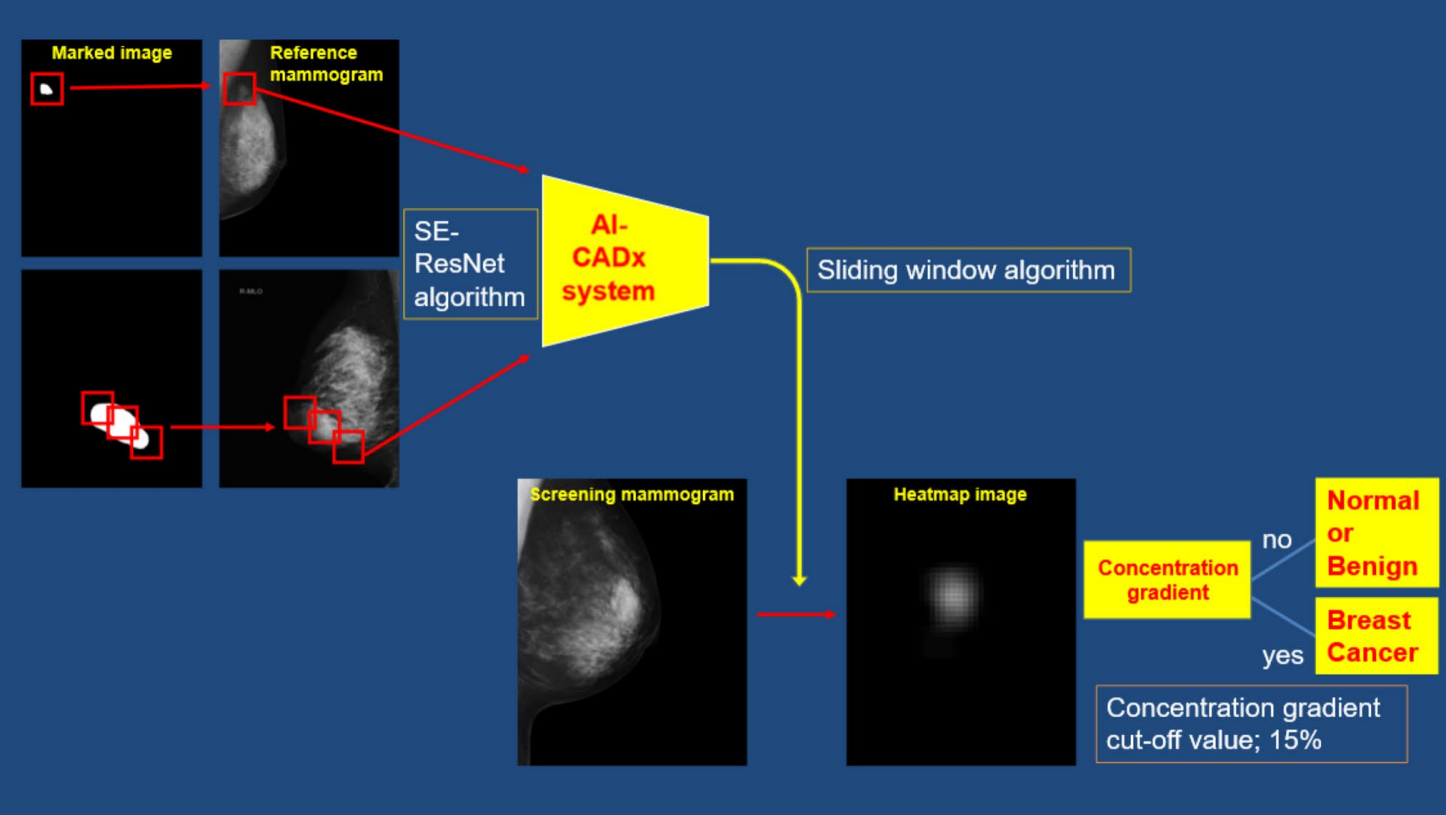
Flow diagram of the AI-CADx system

The flow diagram of our developed AI-CADx system is shown in Fig. 1. We used Python software (version 3.7.7) as the programming language. TensorFlow (version 2.1.0) was used as the AI framework.

The AI architecture is an original design based on a convolutional neural network. First, we formed an squeeze-and-excitation (SE) — residual block by combining convolutional layers, batch normalization layers, and a SE structure. After the residual block, we compressed the data using global average pooling and passed it through two fully connected layers and a sigmoid function to output it as a tensor. The output number indicates the probability of detecting a malignant lesion. During training, the dropout rate was set to 50% and inserted after each fully connected layer. During learning, optimization was performed using reduced focal loss. The parameters were fixed at the recommended values of α = 0.25 and γ = 2.0.

The mammograms used to train the AI-CADx system had different resolutions depending on the manufacturer. To standardize the size of calcifications and other mammographic findings, the mammograms were uniformly resized to a height of 2560 pixels while maintaining the aspect ratio. Then, the mammograms were converted into 8-bit grayscale PNG files with 256 levels before being used for learning. If the original mammogram is 16 bits, for instance, the pixel value is divided by 256 to ensure it falls between 0 and 255.

The gold standard for annotating this AI-CADx system is based on pathologic references. The mammogram was cropped to 512 × 512 pixels based on the marked image so that the detected lesion was sufficiently included. If the lesion was smaller than the cut-out image, the center of gravity of the lesion was calculated from the marked image and set as the center of the cut-out image. If the lesion was larger than the cut-out image, multiple crops were made at equal intervals along the contour of the marked image. This allowed us to create a cut-out image depicting the edge of the lesion and containing approximately 50% of it. During training, patch images were randomly cropped to 384 × 384 pixels and randomly combined using data augmentation techniques, e.g., horizontal flip, filtering (Laplacian, Sobel, Canny edge detection, Gabor, Gaussian, and Median), random shifting, and random erasing. Since we plan to commercialize the current AI-CADx system, we did not perform pretraining on ImageNet. Instead, we trained the model from scratch for 300 epochs.

During testing, the sliding window method was used to detect lesions in a single mammogram. Since lesions may appear at the mammogram’s edges, margins of 384 pixels were added to the top, bottom, left, and right of the mammogram. Lesions were detected using a stride of 64 pixels. If the tensor value was 0.5 or greater, the corresponding area of a prepared heat map image was assigned a probability value. Repeating this process assigned a higher numerical value to areas with a high probability of depicting a lesion. Finally, the heat map image was overlaid on the mammogram to visualize the likelihood of a lesion’s presence (see **Supplementary Fig. 2**).

AI stand-alone was not designed to interpret mammograms independently. Assuming a performance evaluation was conducted on a single unit, areas of the heat map image with a concentration gradient exceeding 15% were identified as having a malignant lesion. This value of 15% was determined through repeated experimentation to strike a good balance between sensitivity and specificity. Using the sliding window method, heat map images were created for all mammograms. If a concentration gradient of 15% or higher was present, the mammogram was determined to contain a malignant lesion (Fig. 1).

### Outcomes

After developing the AI-CADx system with the training and validation datasets, we assessed its performance with an independent test dataset. The test dataset consisted of MLO view mammograms only. The AI-CADx system was considered accurate if it detected the presence of a malignant lesion in a mammogram indicating breast cancer. Localization was not considered in the assessment of accuracy. The Breast Cancer Surveillance Consortium reported radiologist’s interpretive performance at mammography screening with sensitivity and specificity of approximately 85 and 90%, respectively [23]. In addition, the sensitivity of mammography screening was 77% in the J-START study conducted in Japan [24].

Therefore, we set the primary endpoint as the expected sensitivity and specificity of the AI-CADx system for diagnosing breast cancer at 80% or higher.

If the AI-CADx system met this endpoint, we interpreted its performance as equivalent to that of Japanese physicians involved in breast cancer screening, according to the Japan Central Organization on Quality Assurance of Breast Cancer Screening (https://www.qabcs.or.jp/).

We also set the secondary endpoint**s** as follows: the accuracy of the AI-CADx system according to the following subgroups: (i) breast density, (ii) mammographic findings, (iii) pathologic findings, and (iv) mammography vendors.

### Statistical analysis

We evaluated the breast cancer diagnostic performance of the AI-CADx system using the receiver operating characteristic (ROC) curve and the area under the ROC curve (AUC). We calculated the sensitivity, specificity, and AUC of the AI-CADx system at the breast level using the sci-kit learn Python library. Sensitivity is defined as the proportion of mammograms that the AI-CADx system correctly diagnosed breast cancer among breast cancer-positive mammograms. Specificity is the number of mammograms in which the AI-CADx system correctly diagnosed normal or benign lesions among mammograms without breast cancer. Since the sample size was sufficient and the data were expected to follow a standard normal distribution, we used the DeLong method [25] to calculate the 95% confidence interval from the AUC rather than the bootstrap method. All statistical calculations and analyses were performed using Python software (version 3.7.7).

## Results

### Baseline characteristics of the datasets

The baseline characteristics of the datasets are shown in Table 1. We retrospectively collected 20,638 mammograms from 11,450 women. The characteristics were balanced between the training, validation, and test datasets in a ratio of 8:1:1 on a case-level basis (training/validation/test = 9160/1145/1145 cases). Digital radiography mammograms accounted for 91%, and computed radiography mammograms accounted for 9%. The median age of all patients was 55 years. Patients with both MLO and CC view mammograms were available in 76.6%. The distribution of breast density was as follows; almost entirely fatty (14.1%), scattered fibroglandular density (42.9%), heterogeneously dense (30.9%), and extremely dense (10.1%). The median compressed breast thickness was 42 mm. The diagnoses of the collected mammograms were consisted as follows; 5,019 (24.3%) breast cancer, 5,026 (24.4%) benign, and 10,593 (51.3%) normal mammograms. The most common mammographic findings were mass (34.1%), calcification (7.3%), focal asymmetry density (5.1%), and architectural distortion (3.0%).

**Table 1:** Baseline characteristics of the datasets

	All	Training/validation dataset	Test dataset
Number of cases	11,450	10,305	1145
Number of mammograms	20,638	18,579	2059
Median age, years (range)	55 (18—101)	55 (18—101)	56 (21—94)
Type of view – number of mammograms (%)			
Mediolateral oblique only	4829 (23.4%)	4348 (23%)	481 (23%)
Mediolateral oblique and craniocaudal	15,809 (76.6%)	14,231 (77%)	1578 (77%)
Breast density*—number of mammograms (%)			
Entirely fatty (A)	2910 (14.1%)	2604 (14.1%)	306 (14.9%)
(A) or (B) equivocal	132 (0.6%)	121 (0.6%)	11 (0.6%)
Scattered fibroglandular density (B)	8852 (42.9%)	7984 (42.9%)	868 (42.9%)
(B) or (C) equivocal	211 (1.0%)	197 (1.0%)	14 (1.0%)
Heterogeneously dense (C)	6380 (30.9%)	5,758 (30.9%)	622 (30.9%)
(C) or (D) equivocal	69 (0.3%)	58 (0.3%)	11 (0.5%)
Extremely dense (D)	2077 (10.1%)	1,850 (10.1%)	227 (11.0%)
Median compressed breast thickness (mm)	42	42	42
Mammography category*—number of mammograms (%)			
Category 1	10,602 (51%)	9544 (51%)	1058 (51%)
Category 2	1123 (5%)	1008 (5%)	115 (6%)
Category 3	4435 (22%)	3993 (22%)	442 (21%)
Category 4	2,624 (13%)	2363 (13%)	261 (13%)
Category 5	1850 (9%)	1668 (9%)	182 (9%)
Missing data	4 (0%)	3 (0%)	1 (0%)
Diagnosis—number of mammograms (%)			
Breast cancer	5019 (24.3%)	4514 (24.3%)	505 (24.5%)
Benign	5026 (24.4%)	4528 (24.4%)	498 (24.2%)
Normal	10,593 (51.3%)	9,537 (51.3%)	1056 (51.3%)
Mammographic findings†—number of mammograms (%)			
Mass	7046 (34.1%)	6360 (34.2%)	686 (33.3%)
Calcification	1511 (7.3%)	1378 (7.4%)	133 (6.5%)
Focal asymmetry density	1050 (5.1%)	1937 (5.0%)	113 (5.5%)
Architectual distortion	610 (3.0%)	545 (2.9%)	65 (3.2%)
Others	90 (0.4%)	80 (0.4%)	10 (0.5%)
Normal	10,593 (51.3%)	9537 (51.3%)	1056 (51.3%)

Breast density; mammary area with density level equivalent to or higher than the density level of pectoralis major mascle, and the mammary parenchyma area approximately (i) < 10%: Entirely fatty, (ii)10–50%: Scattered fibroglandular density, (iii)50–80%: Heterogeneously dense, (iv) > 80%: Extremely dense

Category 1: negative, 2: benign, 3: benign but malignancy not ruled out, 4: suspicious abnormality, 5: highly suggestive malignancy

*According to the Japanese mammography guidelines 4th edition. (Japan Radiological Society, Japanese Society of Radiological Technology. Mammography guidelines. 4th ed. Igaku-Shoin Ltd. 2021. (in Japanese))

†Because some mammograms had two or more findings, the total does not add up to 100%

### Baseline clinicopathological features of the datasets

The clinicopathological characteristics of the datasets are summarized in Table 2. Among 5,019 breast cancer mammograms, 3,785 (75.4%) had invasive ductal carcinoma, and 595 (11.9%) had ductal carcinoma in situ. Among 5,026 mammograms with benign lesions, 1,391 (27.7%) were histologically diagnosed. The most common benign lesion was fibroadenoma (*n* = 2,573, 51.2%), followed by cyst (*n* = 970, 19.3%), benign calcification (*n* = 657, 13.1%), and mastopathy (*n* = 259, 5.2%).

**Table 2:** Baseline clinicopathological features of the datasets

	All	Training/Validation dataset	Test dataset
Type of breast cancer*†—number of mammograms (%)	5019	4514	505
Invasive ductal carcinoma	3785 (75.4%)	3395 (75.2%)	369 (77.2%)
Ductal carcinoma in situ	595 (11.9%)	536 (11.9%)	59 (11.7%)
Invasive lobular carcinoma	214 (4.3%)	190 (4.2%)	24 (4.8%)
Mucinous carcinoma	204 (4.1%)	187 (4.1%)	17 (3.4%)
Others	251 (5.0%)	232 (5.1%)	19 (3.8%)
Type of benign lesions (biopsy proven)†—number of mammograms (%)	1391	1246	145
Fibroadenoma	762 (54.8%)	679 (54.5%)	83 (57.2%)
Mastopathy	259 (18.6%)	236 (18.9%)	23 (15.9%)
Intra-ductal papilloma	145 (10.4%)	135 (10.8%)	10 (6.9%)
Benign calcification	84 (6.0%)	77 (6.2%)	7 (4.8%)
Adenoma	21 (1.5%)	19 (1.5%)	2 (1.4%)
Others	130 (9.3%)	107 (8.6%)	23 (15.9%)
Type of benign lesions (clinically diagnosed)†—number of mammograms (%)	3635	3282	353
Fibroadenoma	1811 (49.8%)	1639 (49.9%)	172 (48.7%)
Cyst	970 (26.7%)	881 (26.8%)	89 (25.2%)
Benign calcification	573 (15.8%)	518 (15.8%)	55 (15.6%)
Hamartoma	115 (3.2%)	97 (3.0%)	18 (5.1%)
Others	232 (6.4%)	206 (6.3%)	26 (7.4%)

*All cases were confirmed histologically

†Some mammograms had duplicated types

### Breast cancer diagnostic performance of the AI-CADx system

**Fig. 2:**
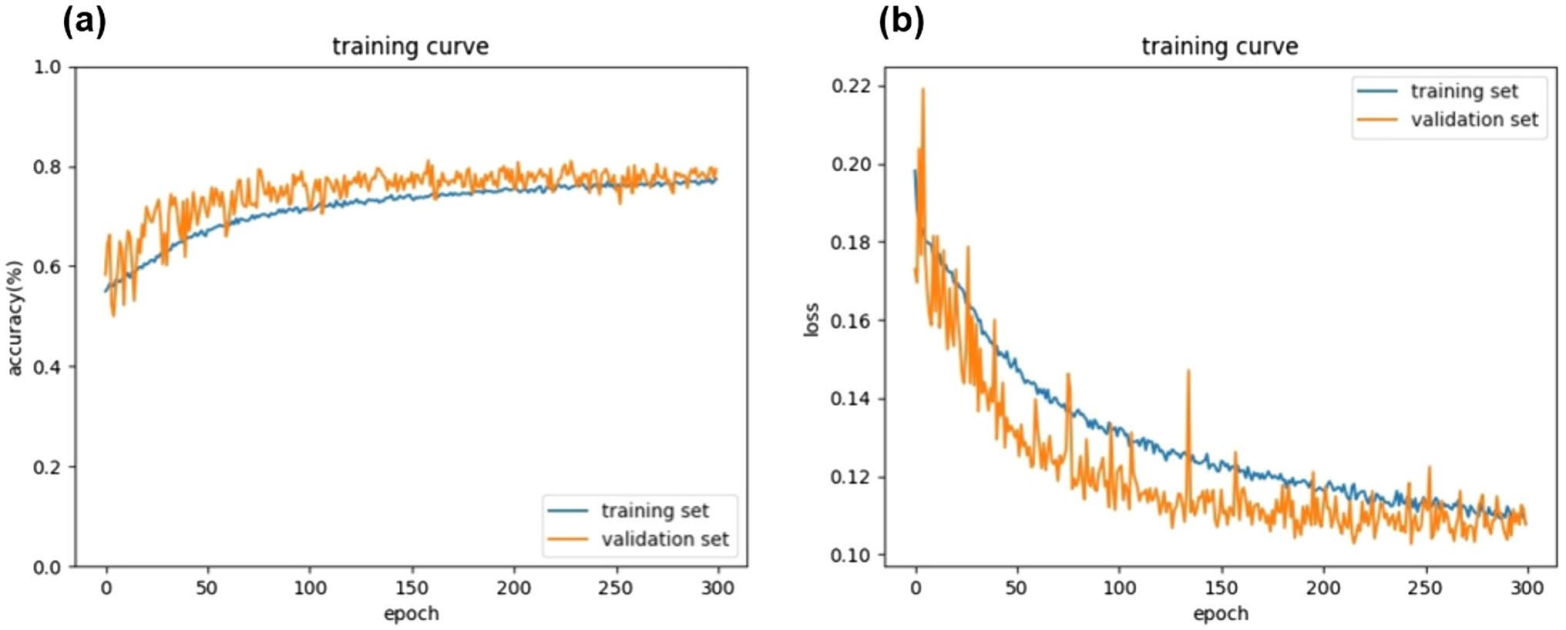
The learning curve of the AI-CADx system in the training process; **a** the accuracy and **b** the loss at that time

The final model of the AI-CADx system was established with the training and validation datasets. The learning curve of the AI-CADx system in the training and validation datasets is shown in Fig. 2. After 300 epochs, the validation dataset, like the training dataset, showed an increase in accuracy and a decrease in loss.

**Fig. 3:**
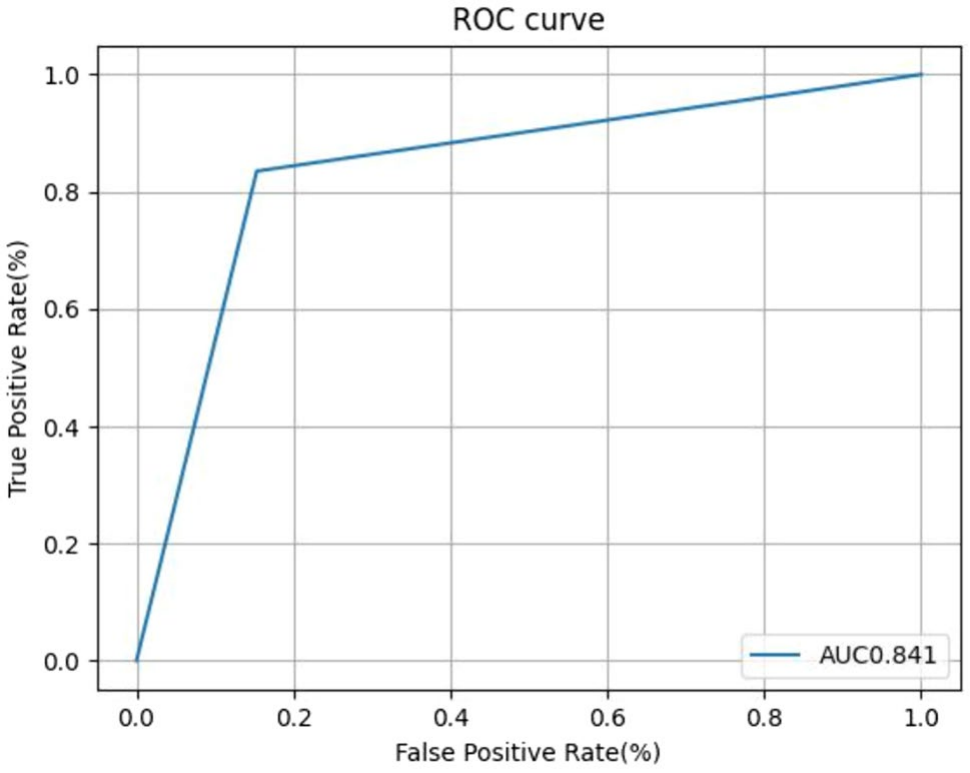
Receiver Operating Curve (ROC) of breast cancer diagnostic performance of the AI-CADx system in the test dataset

In the test dataset (with a breast cancer proportion of 24.5%), the AI-CADx system diagnosed breast cancer with a sensitivity of 83.5% and a specificity of 84.7%, respectively. The accuracy rate was 84.4%. The positive predictive and negative predictive value were 63.8% and 94.1%, respectively. The AUC of the AI-CADx system for breast cancer diagnosis was 0.841 (DeLong 95% CI 0.822–0.859) (Fig. 3).

We further validated the accuracy of the AI-CADx system in the test dataset according to breast density, mammographic findings, pathologic findings, and mammography vendors. Remarkably, our AI-CADx system showed almost consistent accuracy in breast cancer diagnosis independent of these variables (AUC (range); 0.639–0.906) (Table 3).

**Table 3:** Subgroup analysis of breast cancer diagnostic performance of the AI-CADx system in the test dataset (2059 mammograms)

Variables	Sensitivity(%)	Specificity(%)	AUC (DeLong 95%CI)
Test dataset (2059 mammograms)	0.835	0.847	0.841(0.822–0.859)
Breast density*—number of mammograms			
Entirely fatty (*n* = 307)	0.863	0.918	0.890(0.840–0.941)
Scattered fibroglandular density (*n* = 875)	0.871	0.789	0.830(0.804–0.856)
Non-dense(fatty + scattered) (*n* = 1182)	0.87	0.827	0.848(0.826–0.871)
Heterogeneously dense (*n* = 634)	0.806	0.825	0.816(0.781–0.850)
Extremely dense (*n* = 229)	0.421	0.971	0.696(0.582–0.811)
Dense(heterogeneously + extremely dense) (*n* = 863)	0.766	0.87	0.818(0.785–0.852)
Imaging findings*—number of mammograms			
Mass (*n* = 687)	0.848	0.822	0.835(0.807–0.863)
Calcification (*n* = 97)	0.854	0.714	0.784(0.703–0.865)
Focal asymmetry density (*n* = 114)	0.81	0.843	0.826(0.756–0.897)
Architectual distortion (*n* = 67)	0.85	0.429	0.639(0.436–0.842)
Type of breast cancer*—number of mammograms			
Invasive ductal carcinoma (*n* = 372)	0.858	n/a	n/a
Others (*n* = 137)	0.774	n/a	n/a
Type of benign lesions (biopsy proven + clinically diagnosed)*—number of mammograms			
Fibroadenoma (*n* = 83)	n/a	0.759	n/a
Others (*n* = 71)	n/a	0.648	n/a
Mammography vendors*—number of mammograms			
REGIUS MODEL 190 (Konica Minolta, Japan) (*n* = 32)	0.875	0.813	0.844(0.714–0.973)
FCR PROFECT CS (FUJIFILM, Japan) (*n* = 125)	0.769	0.92	0.844(0.723–0.966)
Senographe-Crystal Nova, Senographe-Pristina, Senographe-Essential (GE HealthCare Technologies, USA) (*n* = 196)	0.855	0.731	0.793(0.735–0.851)
Pe・ru・ru DIGITAL, Pe・ru・ru LaPlus (Canon Medical Systems, Japan) (*n* = 28)	1	0.813	0.906(0.807–1.000)
MAMMOMAT-Inspiration, MAMMOMAT-Revelation (Siemens Healthineers, Germany) (*n* = 400)	0.736	0.916	0.826(0.778–0.874)
Selenia-Dimensions (Hologic, USA) (*n* = 847)	0.877	0.837	0.857(0.830–0.884)
AMULET-f, AMULET-Innovality (FUJIFILM, Japan) (*n* = 453)	0.811	0.827	0.819(0.777–0.861)

*AI-CADx* Artificial Intelligence – computer-aided diagnosis, *AUC* Area under the receiver operating characteristic curve, *95%CI* 95% confidence interval, *n/a* not available

*The number of mammograms may differ due to validation purposes

## Discussion

In this study, we successfully developed the mammographic AI-CADx system specifically designed for Japanese women. This system demonstrated pre-specified breast cancer diagnostic accuracy, suggesting its expectation as a supporting tool for breast cancer screening in Japan.

Compared to Western women, Japanese women have a higher prevalence of dense breasts [26]. To address this challenge, our study developed the AI-CADx system, which is adapted to the unique characteristics of Japanese women. Indeed, the present AI-CADx system showed consistent diagnostic accuracy for breast cancer independent of breast density. In addition, mammographic findings, e.g., mass and calcification were detected without bias across the range of breast density. Furthermore, the strength of our AI-CADx system lies in its composition of multi-vendor mammograms, which demonstrates its high versatility. In fact, the AI-CADx system’s accuracy was nearly consistent across all vendors.

We prioritized quality in the generation process of the training data. Experienced investigators interpreted the mammograms and outlined the malignant and benign lesions. The diagnosis was confirmed by histopathology. And benign lesions without pathologic references were diagnosed clinically at least two years apart. This meticulous approach allowed us to create a reliable dataset. Actually, our AI-CADx system correctly identified malignant lesions regardless of breast cancer type or benign lesion. However, there are some caveats. There is a possibility that breast cancers that are not visible on a mammogram may not be detected as well as they would be with human interpretation. But if the features appear on the mammogram, our AI-CADx system may be able to detect them, even if they are not visible to the human eye.

To date, no other large-scale mammographic AI-CADx system has been developed in Japan. Ueda et al. developed the AI-CADe system with 4,636 mammograms in Japan [27]. The accuracy of breast cancer detection with an AUC of 0.93 in test datasets is expected to serve as a first reader in Japan. In fact, reports indicate that stand-alone overseas mammographic AI-CADe systems show inferior diagnostic performance compared to those of Japanese readers [28]. This suggests that a decrease in accuracy may be a race-related issue [28]. We plan to conduct a prospective clinical trial (UMIN, trial number UMIN000049479. Registered 11 November 2022, https://www.umin.ac.jp/ctr/) in future. The trial aims to verify whether our AI-CADx system can improve the breast cancer diagnostic performance of Japanese physicians on mammography readings. This study will identify which populations will benefit from the AI-CADx system by collecting the AUC from 30 Japanese physicians who have different levels of mammography reading experience. If our AI-CADx system is found to be beneficial as a second or concurrent reader for inexperienced mammography readers, we believe that adopting our AI-CADx system will improve the overall accuracy of breast cancer screening in Japan.

This study has some limitations. First, we were unable to evaluate the breast cancer diagnostic performance of our AI-CADx system according to molecular subtypes. However, when considering imaging characteristics by molecular subtypes, the consistent accuracy in identifying mammographic findings, e.g., mass or calcification, indicates the versatility of this AI-CADx system. Second, since the data were collected primarily from 63 specific institutions in Japan, our findings may not be generalizable to other regions. Finally, our AI-CADx system was designed for Japanese women, so its applicability to other countries has not been confirmed. However, we expect our AI-CADx system to perform well for Asian women who have similar breast density, size, and shape similar to that of Japanese women.

In conclusion, we have developed a mammographic AI-CADx system for Japanese women for the first time through a large-scale multi-institutional clinical trial. We expect our planned prospective study to determine the potential of this AI-CADx system as a second reader for Japanese physicians.

## Supplementary Information

Below is the link to the electronic supplementary material.

Supplementary file1 Mediolateral oblique (MLO) views of breast cancer or benign lesion cases, and their marked 26 images by Fiji software; (a) mass, (b) calcification, (c) focal asymmetry density, (d) architectural distortion, and (e) fibroadenoma. (JPG 84 KB)

Supplementary file2 Mediolateral oblique (MLO) view of left breast cancer (a), and heatmap image of automated breast cancer diagnosis by the AI-CADx system (b). (JPG 45 KB)

## Data Availability

The datasets used in the current study are available from the corresponding author upon reasonable request.

## Abbreviations

CADe: Computer aided detection,
AI: Artificial intelligence,
CADx: Computer aided diagnosis,
CNN: Convolutional neural networks,
MLO: Medio-lateral oblique,
CC: Cranio-caudal,
AUC: Area under the receiver operating characteristic curve

## References

[1] Nelson HD, Tyne K, Naik A, Bougatsos C, Chan BK, Humphrey L, et al. Screening for breast cancer: an update for the U.S. Preventive services task force. Ann Intern Med. 2009;151(727–37):W237–42. 10.1059/0003-4819-151-10-200911170-00009 PMC2972726 19920273

[2] Independent UKPoBCS. The benefits and harms of breast cancer screening: an independent review. Lancet. 2012;380:1778–86. 23117178 10.1016/S0140-6736(12)61611-0

[3] Miller AB, Wall C, Baines CJ, Sun P, To T, Narod SA. Twenty five year follow-up for breast cancer incidence and mortality of the Canadian national breast screening study: randomised screening trial. BMJ. 2014;348: g366. 24519768 10.1136/bmj.g366 PMC3921437

[4] Kubota K, Nakashima K, Nakashima K, Kataoka M, Inoue K, Goto M, et al. The Japanese breast cancer society clinical practice guidelines for breast cancer screening and diagnosis, 2022 edition. Breast Cancer. 2024;31:157–64. 37973686 10.1007/s12282-023-01521-x PMC10901949

[5] Taylor P, Potts HW. Computer aids and human second reading as interventions in screening mammography: two systematic reviews to compare effects on cancer detection and recall rate. Eur J Cancer. 2008;44:798–807. 18353630 10.1016/j.ejca.2008.02.016

[6] Miglioretti DL, Smith-Bindman R, Abraham L, Brenner RJ, Carney PA, Bowles EJ, et al. Radiologist characteristics associated with interpretive performance of diagnostic mammography. J Natl Cancer Inst. 2007;99:1854–63. 18073379 10.1093/jnci/djm238 PMC3144707

[7] Elmore JG, Jackson SL, Abraham L, Miglioretti DL, Carney PA, Geller BM, et al. Variability in interpretive performance at screening mammography and radiologists’ characteristics associated with accuracy. Radiology. 2009;253:641–51. 19864507 10.1148/radiol.2533082308 PMC2786197

[8] Freer TW, Ulissey MJ. Screening mammography with computer-aided detection: prospective study of 12,860 patients in a community breast center. Radiology. 2001;220:781–6. 11526282 10.1148/radiol.2203001282

[9] Cupples TE, Cunningham JE, Reynolds JC. Impact of computer-aided detection in a regional screening mammography program. AJR Am J Roentgenol. 2005;185:944–50. 16177413 10.2214/AJR.04.1300

[10] Morton MJ, Whaley DH, Brandt KR, Amrami KK. Screening mammograms: interpretation with computer-aided detection–prospective evaluation. Radiology. 2006;239:375–83. 16569779 10.1148/radiol.2392042121

[11] Gilbert FJ, Astley SM, Gillan MG, Agbaje OF, Wallis MG, James J, et al. Single reading with computer-aided detection for screening mammography. N Engl J Med. 2008;359:1675–84. 18832239 10.1056/NEJMoa0803545

[12] Fenton JJ, Taplin SH, Carney PA, Abraham L, Sickles EA, D’Orsi C, et al. Influence of computer-aided detection on performance of screening mammography. N Engl J Med. 2007;356:1399–409. 17409321 10.1056/NEJMoa066099 PMC3182841

[13] Fenton JJ, Abraham L, Taplin SH, Geller BM, Carney PA, D’Orsi C, et al. Effectiveness of computer-aided detection in community mammography practice. J Natl Cancer Inst. 2011;103:1152–61. 21795668 10.1093/jnci/djr206 PMC3149041

[14] Lehman CD, Wellman RD, Buist DS, Kerlikowske K, Tosteson AN, Miglioretti DL, et al. Diagnostic accuracy of digital screening mammography with and without computer-aided detection. JAMA Intern Med. 2015;175:1828–37. 26414882 10.1001/jamainternmed.2015.5231 PMC4836172

[15] Rodriguez-Ruiz A, Krupinski E, Mordang JJ, Schilling K, Heywang-Kobrunner SH, Sechopoulos I, et al. Detection of breast cancer with mammography: effect of an artificial intelligence support system. Radiology. 2019;290:305–14. 30457482 10.1148/radiol.2018181371

[16] Kim HE, Kim HH, Han BK, Kim KH, Han K, Nam H, et al. Changes in cancer detection and false-positive recall in mammography using artificial intelligence: a retrospective, multireader study. Lancet Digit Health. 2020;2:e138–48. 33334578 10.1016/S2589-7500(20)30003-0

[17] Pacilè S, Lopez J, Chone P, Bertinotti T, Grouin JM, Fillard P. Improving breast cancer detection accuracy of mammography with the concurrent use of an artificial intelligence tool. Radiol Artif Intell. 2020;2: e190208. 33937844 10.1148/ryai.2020190208 PMC8082372

[18] Salim M, Wåhlin E, Dembrower K, Azavedo E, Foukakis T, Liu Y, et al. Strand F. external evaluation of 3 commercial artificial intelligence algorithms for independent assessment of screening mammograms. JAMA Oncol. 2020;6:1581–8. 32852536 10.1001/jamaoncol.2020.3321 PMC7453345

[19] Ishihara S, Taira N, Kawasaki K, Ishibe Y, Mizoo T, Nishiyama K, et al. Association between mammographic breast density and lifestyle in Japanese women. Acta Med Okayama. 2013;67:145–51. 23804137 10.18926/AMO/50407

[20] Bae JM, Shin SY, Kim EH, Kim YN, Nam CM. Distribution of dense breasts using screening mammography in Korean women: a retrospective observational study. Epidemiol Health. 2014;36: e2014027. 25381996 10.4178/epih/e2014027 PMC4258717

[21] Dai H, Yan Y, Wang P, Liu P, Cao Y, Xiong L, et al. Distribution of mammographic density and its influential factors among Chinese women. Int J Epidemiol. 2014;43:1240–51. 24639441 10.1093/ije/dyu042 PMC4121553

[22] Burt JR, Torosdagli N, Khosravan N, RaviPrakash H, Mortazi A, Tissavirasingham F, et al. Deep learning beyond cats and dogs: recent advances in diagnosing breast cancer with deep neural networks. Br J Radiol. 2018;91:20170545. 29565644 10.1259/bjr.20170545 PMC6223155

[23] Ichikawa LE, Barlow WE, Anderson ML, Taplin SH, Geller BM, Brenner RJ, et al. Time trends in radiologists’ interpretive performance at screening mammography from the community-based Breast Cancer Surveillance Consortium, 1996–2004. Radiology. 2010;256:74–82. 20505059 10.1148/radiol.10091881 PMC2897687

[24] Ohuchi N, Suzuki A, Sobue T, Kawai M, Yamamoto S, Zheng YF, et al. Sensitivity and specificity of mammography and adjunctive ultrasonography to screen for breast cancer in the Japan Strategic Anti-cancer Randomized Trial (J-START): a randomised controlled trial. Lancet. 2016;387:341–8. 26547101 10.1016/S0140-6736(15)00774-6

[25] DeLong ER, DeLong DM, Clarke-Pearson DL. Comparing the areas under two or more correlated receiver operating characteristic curves: a nonparametric approach. Biometrics. 1988;44:837–45. 3203132

[26] Tice JA, Cummings SR, Smith-Bindman R, Ichikawa L, Barlow WE, Kerlikowske K. Using clinical factors and mammographic breast density to estimate breast cancer risk: development and validation of a new predictive model. Ann Intern Med. 2008;148:337–47. 18316752 10.7326/0003-4819-148-5-200803040-00004 PMC2674327

[27] Ueda D, Yamamoto A, Onoda N, Takashima T, Noda S, Kashiwagi S, et al. Development and validation of a deep learning model for detection of breast cancers in mammography from multi-institutional datasets. PLoS ONE. 2022;17: e0265751. 35324962 10.1371/journal.pone.0265751 PMC8947392

[28] Sasaki M, Tozaki M, Rodríguez-Ruiz A, Yotsumoto D, Ichiki Y, Terawaki A, et al. Artificial intelligence for breast cancer detection in mammography: experience of use of the screenpoint medical transpara system in 310 Japanese women. Breast Cancer. 2020;27:642–51. 32052311 10.1007/s12282-020-01061-8

