# Supplementary figures and images for: Development of a deep learning-based automated diagnostic system (DLADS) for classifying mammographic lesions — a first large-scale multi-institutional clinical trial in Japan
Source: Breast Cancer. 2025 Jul 3;32(5):1115–24. doi: 10.1007/s12282-025-01741-3 (PMC12394324; doi:10.1007/s12282-025-01741-3)

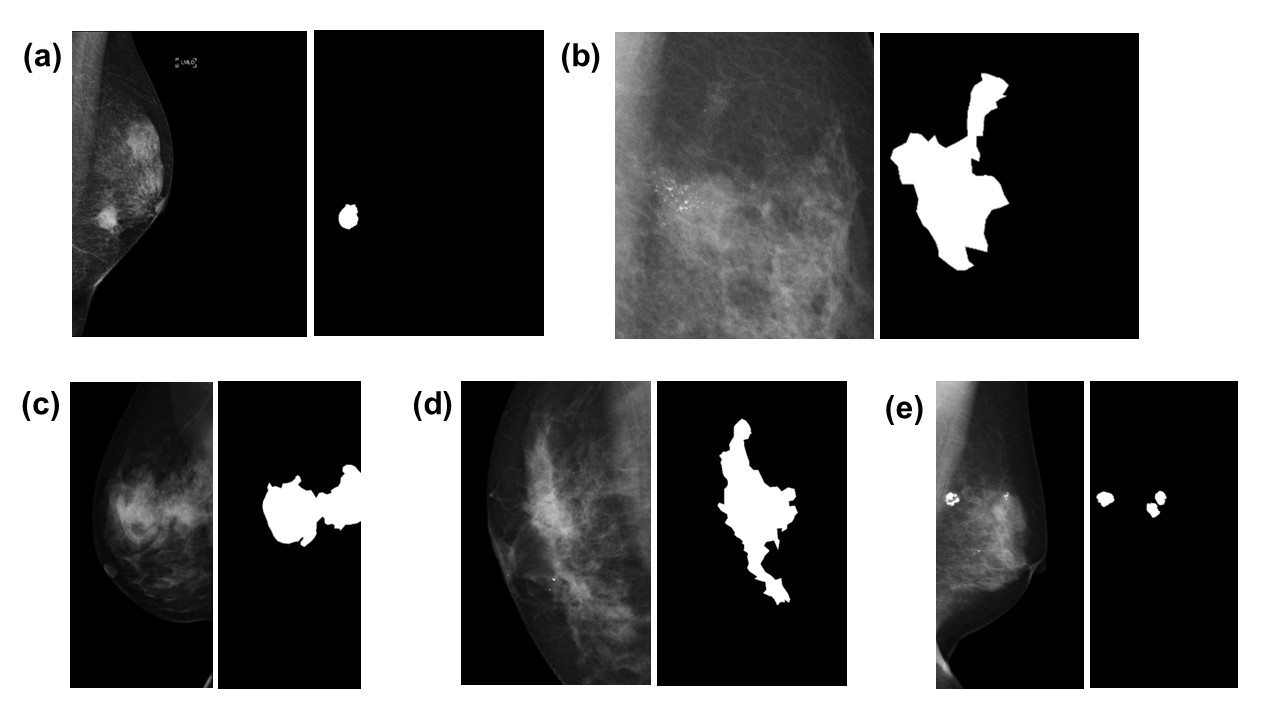

Supplement: Supplementary file 1 — Supplementary file1 Mediolateral oblique (MLO) views of breast cancer or benign lesion cases, and their marked 26 images by Fiji software; (a) mass, (b) calcification, (c) focal asymmetry density, (d) architectural distortion, and (e) fibroadenoma. (JPG 84 KB) [file 12282_2025_1741_MOESM1_ESM.jpg]

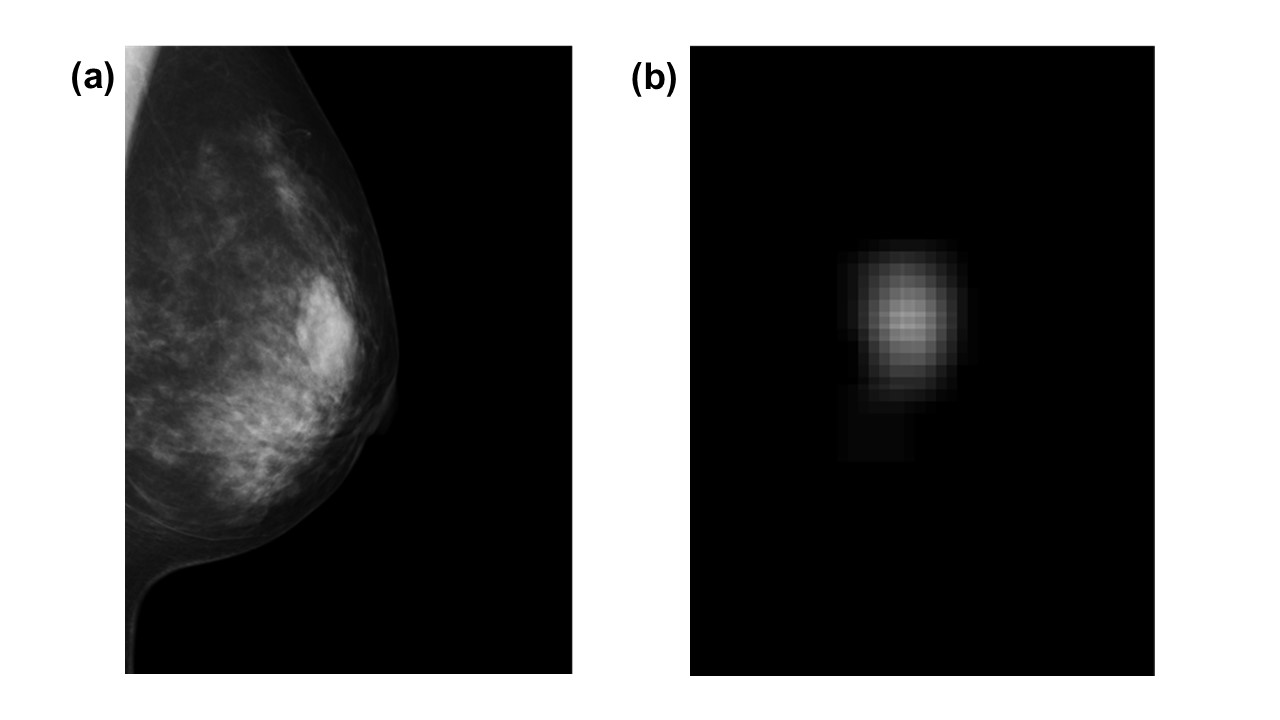

Supplement: Supplementary file 2 — Supplementary file2 Mediolateral oblique (MLO) view of left breast cancer (a), and heatmap image of automated breast cancer diagnosis by the AI-CADx system (b). (JPG 45 KB) [file 12282_2025_1741_MOESM2_ESM.jpg]
